# Clinical Utility of Metagenomic Next-Generation Sequencing in Adult Patients with Fever of Unknown Origin: A Retrospective Real-World Study

**DOI:** 10.3390/jcm15156038

**Published:** 2026-08-03

**Authors:** Fuping Guo, Li Zhang, Zhengyin Liu, Baotong Zhou, Hongwei Fan, Dong Zhang, Qiwen Yang, Taisheng Li, Ying Ge

**Affiliations:** 1 Department of Infectious Diseases, Peking Union Medical College Hospital, Chinese Academy of Medical Sciences, Beijing 100730, China; guofp@pumch.cn (F.G.);; 2 Department of Laboratory Medicine, Peking Union Medical College Hospital, Chinese Academy of Medical Sciences, Beijing 100730, China

**Keywords:** fever of unknown origin, metagenomic next-generation sequencing, occult infection, tuberculosis, diagnostic utility, infectious diseases

## Abstract

**Background:** Fever of unknown origin (FUO) remains a major diagnostic challenge due to its heterogeneous etiologies and nonspecific clinical manifestations. Although metagenomic next-generation sequencing (mNGS) represents a promising diagnostic tool, its clinical utility in adult patients with FUO remains incompletely characterized. **Methods:** In this study, we retrospectively analyzed adult FUO patients who underwent mNGS testing at Peking Union Medical College Hospital between March 2022 and April 2024. Clinically meaningful diagnostic contribution was determined according to the final clinical diagnosis following multidisciplinary adjudication. Diagnostic performance, pathogen spectrum, therapeutic impact, specimen type, and predictors of clinically meaningful mNGS results were evaluated. **Results:** A total of 127 FUO patients were included in the study. Infectious diseases accounted for 53.5% of final diagnoses, followed by noninfectious inflammatory diseases (11.0%), malignancies (10.2%), and undiagnosed conditions (19.7%). mNGS made a clinically meaningful diagnostic contribution in 31.5% (40/127) of patients, despite an overall positivity rate of 56.7% (72/127), and showed a higher sensitivity than conventional culture for infectious etiologies (69.1% vs. 16.9%), though with a lower specificity (57.6% vs. 96.0%). Diagnostic contribution varied significantly by specimen type, with drainage fluid/abscess samples showing the highest diagnostic yield (90.9%). Lower white blood cell count was independently associated with clinically meaningful mNGS results (OR 0.87, 95% CI 0.77–0.98). **Conclusions:** mNGS provides clinically meaningful diagnostic value in adult patients with FUO, particularly for identifying occult infectious etiologies. Lesion-directed sampling, whenever feasible, and careful interpretation of sequencing results in the clinical context are essential to maximize the diagnostic utility of this approach. A lower white blood cell count was independently associated with clinically meaningful mNGS results, although this finding requires validation in larger prospective studies.

## 1. Introduction

Fever of unknown origin (FUO) remains one of the most challenging clinical syndromes despite substantial advances in laboratory testing and imaging technologies. Classical FUO is defined as a body temperature exceeding 38.3 °C on multiple occasions for more than three weeks without a definitive diagnosis after appropriate evaluation [1,2]. The etiological spectrum of FUO is broad and includes infectious diseases, noninfectious inflammatory diseases (NIIDs), malignancies, and miscellaneous disorders [3]. Although diagnostic approaches have improved considerably, establishing an etiological diagnosis remains difficult in a substantial proportion of patients.

Infectious diseases remain one of the leading causes of FUO [2,4], particularly in developing countries. Nevertheless, microbiological diagnosis is often challenging due to uncommon, fastidious, intracellular, or difficult-to-culture pathogens, as well as atypical clinical manifestations. Conventional microbiological methods, including culturing, serological assays, and targeted polymerase chain reaction (PCR), are limited by suboptimal sensitivity and their reliance on predefined diagnostic hypotheses.

Metagenomic next-generation sequencing (mNGS) has emerged as a promising diagnostic approach that enables the unbiased detection of microbial nucleic acids directly from clinical specimens without prior assumptions regarding the causative pathogen [5,6]. Previous studies have demonstrated the value of mNGS in severe pneumonia, bloodstream infections, and central nervous system infections [7,8,9,10], suggesting particular potential for patients with FUO [11,12].

Despite increasing clinical application, evidence regarding the utility of mNGS in FUO remains limited. Most of the published studies have focused primarily on pathogen detection rates [8,11,12,13] rather than the clinically meaningful diagnostic contributions of mNGS or its impact on patient management. In addition, the influence of specimen type and the factors associated with clinically meaningful mNGS results have not been fully elucidated. Therefore, we conducted a retrospective study to evaluate the real-world clinical utility of mNGS in adult patients with FUO, focusing on its clinically meaningful diagnostic contribution, diagnostic performance, specimen-specific diagnostic yield, therapeutic impact, and factors associated with clinically meaningful mNGS results.

## 2. Materials and Methods

### 2.1. Study Design and Patient Selection

**Figure 1:**
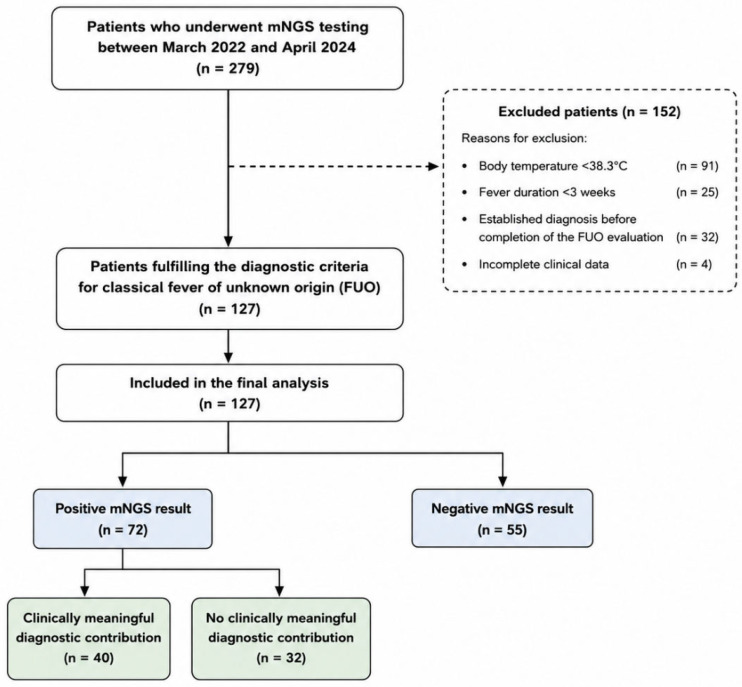
Study flow diagram of patient selection and mNGS results. During the study period, 279 adult patients underwent metagenomic next-generation sequencing (mNGS). After application of the predefined inclusion and exclusion criteria, 127 patients fulfilled the diagnostic criteria for classical fever of unknown origin (FUO) and were included in the final analysis. Among these patients, 72 had positive mNGS results, of which 40 demonstrated a clinically meaningful diagnostic contribution.

This retrospective observational study was conducted at the Department of Infectious Diseases, Peking Union Medical College Hospital (PUMCH)—a national tertiary referral center in China. Between March 2022 and April 2024, 279 adult patients who underwent metagenomic next-generation sequencing (mNGS) were retrospectively screened. After the application of the predefined inclusion and exclusion criteria, 127 patients fulfilled the diagnostic criteria for classical fever of unknown origin (FUO) and were included in the final analysis. The 152 excluded patients did not meet the diagnostic criteria for classical FUO or had incomplete clinical data (Figure 1).

Classical FUO was defined as (1) body temperature >38.3 °C on several occasions, (2) fever duration >3 weeks, and (3) no diagnosis despite appropriate evaluation. Patients younger than 18 years or with incomplete clinical data were excluded.

This study was approved by the Ethics Committee of Peking Union Medical College Hospital. The requirement for informed consent was waived due to the retrospective study design.

### 2.2. Data Collection and Diagnostic Classification

Clinical data were extracted from electronic medical records, including demographic characteristics, comorbidities, immunocompromised status, laboratory findings, specimen type, conventional microbiological results, mNGS findings, final diagnosis, and length of hospital stay.

Immunocompromised status was defined as the presence of hematologic malignancy, solid organ transplantation, neutropenia, human immunodeficiency virus infection, or receipt of long-term corticosteroid or immunosuppressive therapy.

Final diagnoses were established using clinical manifestations, laboratory findings, microbiological investigations, imaging, histopathology, treatment response, and follow-up data. Diagnoses were categorized as infectious diseases, NIIDs, malignancies, miscellaneous disorders, or undiagnosed diseases. Two experienced infectious disease physicians independently reviewed the clinical significance of all mNGS results according to predefined criteria, and any disagreements were resolved through consensus discussion.

### 2.3. mNGS Testing and Clinical Interpretation

mNGS was performed on plasma, bronchoalveolar lavage fluid (BALF), cerebrospinal fluid (CSF), tissue, serous cavity fluid, and drainage fluid specimens by the Clinical Laboratory Department of PUMCH using the Illumina NextSeq CN500 platform (Illumina, San Diego, CA, USA). Sample processing, nucleic acid extraction, library preparation, sequencing, quality control, bioinformatic analyses, and pathogen reporting were conducted according to previously published protocols and are therefore not repeated here in detail [14].

The clinically meaningful diagnostic contribution of mNGS was evaluated according to the final clinical diagnosis. An mNGS result was considered to have a clinically meaningful diagnostic contribution when the detected pathogen was consistent with the patient’s clinical presentation, radiological findings, treatment response, and final diagnosis. Results interpreted as contaminants, colonizing microorganisms, latent viral reactivation, or microorganisms unrelated to the final diagnosis were considered non-contributory.

### 2.4. Assessment of Diagnostic Performance

The diagnostic performance of mNGS and conventional culture in identifying infectious etiologies was evaluated using the final clinical diagnosis as the reference standard. Sensitivity, specificity, positive predictive value (PPV), and negative predictive value (NPV) were calculated. The 95% confidence intervals (CIs) for sensitivity, specificity, positive predictive value (PPV), and negative predictive value (NPV) were calculated using the exact Clopper–Pearson binomial method.

### 2.5. Statistical Analysis

Continuous variables are presented as mean ± standard deviation (SD) or median with interquartile range (IQR), as appropriate, whereas categorical variables are presented as frequencies and percentages. Comparisons between groups were performed using Student’s *t*-test and the Mann–Whitney *U* test for continuous variables or the chi-square test and Fisher’s exact test for categorical variables, as appropriate.

Variables with *p* < 0.10 in univariable analyses, together with clinically relevant variables, were entered into a multivariable logistic regression model to identify factors associated with clinically meaningful mNGS results. Odds ratios (ORs) and 95% confidence intervals (CIs) were calculated.

All statistical tests were two-sided, and *p* < 0.05 was considered statistically significant. Statistical analyses were performed using IBM SPSS Statistics version 26.0 (IBM Corp., Armonk, NY, USA).

## 3. Results

### 3.1. Patient Characteristics and Etiological Distribution

After the application of the predefined eligibility criteria (Figure 1), 127 patients with classical FUO were included in the final analysis. The patients’ baseline characteristics are summarized in Table 1. Infectious diseases accounted for 53.5% of final diagnoses, followed by undiagnosed diseases (19.7%), NIIDs (11.0%), malignancies (10.2%), and miscellaneous disorders (5.5%).

### 3.2. Clinically Meaningful Diagnostic Contribution of mNGS

Among the 127 patients with FUO, mNGS yielded positive results in 72 cases, corresponding to an overall positivity rate of 56.7%. Of these, mNGS made a clinically meaningful diagnostic contribution in 40 patients, representing 31.5% of the entire cohort and 55.6% of patients with positive mNGS findings. The remaining 32 positive results were considered clinically non-contributory (Figure 1).

### 3.3. Clinically Meaningful Diagnostic Contribution According to Specimen Type

**Figure 2:**
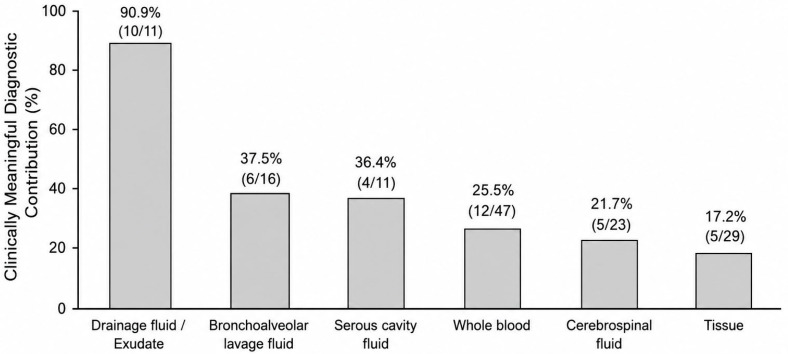
Clinically meaningful diagnostic contribution of mNGS according to specimen type in patients with fever of unknown origin (FUO). Values above each bar indicate the percentage and the corresponding numerator/denominator (contributory cases/total specimens). Abbreviations: mNGS, metagenomic next-generation sequencing.

The rate of clinically meaningful diagnostic contribution varied significantly according to specimen type (Figure 2). Drainage fluid/exudate specimens showed the highest clinically meaningful diagnostic contribution (90.9%), followed by BALF and serous cavity fluid, whereas plasma, CSF, and tissue specimens showed relatively lower diagnostic performance.

### 3.4. Pathogen Spectrum Identified by mNGS

**Figure 3:**
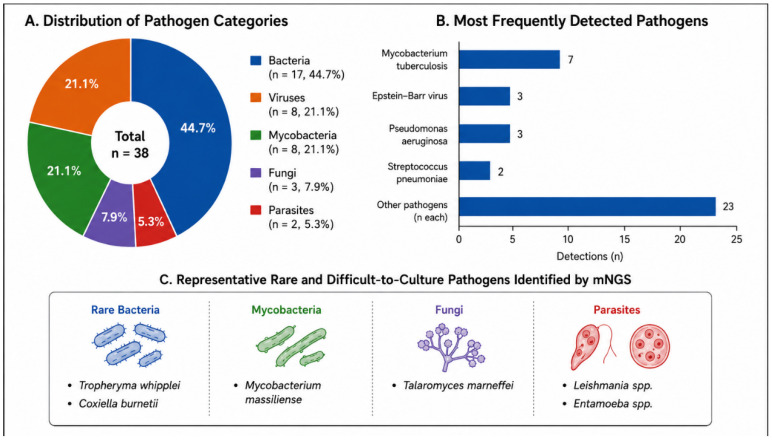
Distribution of pathogens identified by metagenomic next-generation sequencing (mNGS) in patients with fever of unknown origin (FUO). (**A**) Distribution of pathogen categories. (**B**) Most frequently detected pathogens. (**C**) Representative rare and difficult-to-culture pathogens identified by mNGS.

Among the 40 patients with clinically meaningful mNGS results, a total of 38 clinically relevant pathogens were identified (Figure 3). Bacteria represented the largest pathogen category (44.7%), followed by viruses (21.1%) and mycobacteria (21.1%).

Mycobacterium tuberculosis was the most frequently detected pathogen (n = 7), while Epstein–Barr virus and Pseudomonas aeruginosa were each identified in three cases.

In addition, mNGS identified several uncommon or difficult-to-culture pathogens, including *Tropheryma whipplei*, *Coxiella burnetii*, *Leishmania* spp., *Entamoeba* spp., *Talaromyces marneffei*, and Mycobacterium massiliense.

### 3.5. Diagnostic Performance of mNGS and Conventional Culture

Compared with conventional culture, mNGS showed a substantially higher sensitivity (69.1% vs. 16.9%) but lower specificity (57.6% vs. 96.0%) for identifying infectious etiologies (Table 2).

### 3.6. Clinical Impact of mNGS on Antimicrobial Management

**Figure 4:**
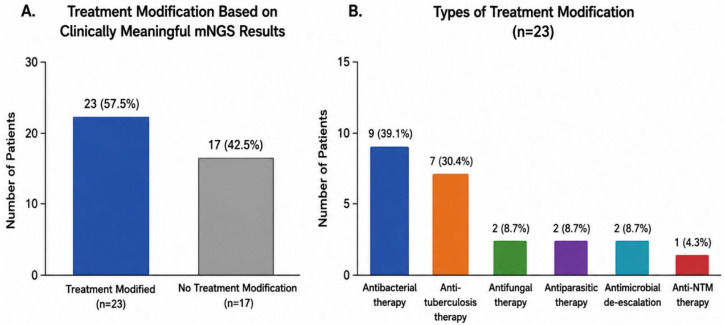
Clinical impact of clinically meaningful mNGS results on treatment decisions in patients with FUO. (**A**) Proportion of patients with clinically meaningful mNGS results who experienced treatment modification. (**B**) Types of treatment modification among the 23 patients with treatment changes. Percentages in panel B are calculated based on the 23 patients with treatment modification. Abbreviations: mNGS, metagenomic next-generation sequencing; FUO, fever of unknown origin; NTM, nontuberculous mycobacteria.

Among the 40 patients for whom mNGS made a clinically meaningful diagnostic contribution (Figure 4), 23 (57.5%) experienced treatment modification based on the mNGS results. Treatment modification consisted primarily of the initiation of targeted antibacterial therapy (n = 9) and anti-tuberculosis therapy (n = 7), with smaller numbers receiving antifungal, antiparasitic, or anti-nontuberculous mycobacterial therapy. Antimicrobial de-escalation occurred in two patients. In the remaining 17 patients (42.5%), mNGS results did not lead to treatment modification because the identified organisms were considered clinically covered, colonizing, or not requiring specific therapy.

### 3.7. Association Between Negative mNGS Results and Noninfectious Etiologies

Among patients with negative mNGS findings, 34 of 55 (61.8%) were ultimately diagnosed with noninfectious diseases, compared with 25 of 72 (34.7%) among those with positive mNGS results. Negative mNGS results were associated with a higher likelihood of a final noninfectious diagnosis (OR 3.04, 95% CI 1.47–6.31; *p* = 0.003).

### 3.8. Factors Associated with Clinically Meaningful Diagnostic Contribution of mNGS

In univariable analyses, younger age, lower WBC count, and lower AST levels were associated with clinically meaningful mNGS results (Table 3). Age, WBC, AST, and PCT were entered into the multivariable logistic regression model. A lower WBC count was independently associated with clinically meaningful mNGS results (OR 0.87, 95% CI 0.77–0.98, *p* = 0.02), whereas age and AST showed borderline associations (Table 4).

## 4. Discussion

In this retrospective study, we evaluated the clinical utility of mNGS in adult patients with fever of unknown origin. Although the overall positivity rate of mNGS was 56.7%, a clinically meaningful diagnostic contribution was obtained in only 31.5% of patients, emphasizing that sequencing results should always be interpreted in the appropriate clinical context. Compared with conventional culture, mNGS demonstrated substantially higher sensitivity for identifying infectious etiologies, supporting its complementary role in the diagnostic evaluation of FUO.

A notable finding was that mNGS influenced clinical management in more than half of patients with clinically meaningful mNGS results. Among the 40 patients with clinically meaningful mNGS findings, antimicrobial therapy was modified in 23 (57.5%), most commonly through the initiation of targeted antibacterial or anti-tuberculosis treatment. Similar management changes have been reported in previous FUO studies, suggesting that the clinical value of mNGS extends beyond pathogen identification to facilitating earlier and more individualized antimicrobial therapy [13,15,16].

Consistent with previous epidemiological studies, infectious diseases remained the leading cause of FUO in our cohort (53.5%) [4,17]. Meanwhile, Mycobacterium tuberculosis was among the most frequently detected pathogens, indicating that occult tuberculosis continues to represent an important cause of FUO in China [18]. In addition, mNGS identified several diagnostically challenging pathogens, including *Tropheryma whipplei*, *Coxiella burnetii*, *Leishmania* spp., and *Talaromyces marneffei*, highlighting its value for detecting uncommon or difficult-to-culture microorganisms that may be missed by conventional microbiological methods [19,20,21,22].

Interestingly, negative mNGS results also provided clinically useful information. In our cohort, patients with negative mNGS results were significantly more likely to receive a final noninfectious diagnosis than those with positive findings. Although a negative mNGS result cannot definitively exclude infection, the high sensitivity of mNGS for infectious etiologies suggests that a negative result may lower the probability of an underlying infectious cause and support further evaluation for autoimmune diseases, malignancies, and other inflammatory disorders [13,15].

The viral pathogens detected in our cohort were predominantly Epstein–Barr virus (EBV) and cytomegalovirus (CMV). Because herpesvirus reactivation is common in chronic inflammation and immune dysregulation, the detection of viral nucleic acids does not necessarily indicate active disease [5]. Therefore, viral mNGS results should be interpreted together with viral load, host immune status, imaging findings, and the overall clinical presentation. Consistent with previous reports [5,23], some positive mNGS findings were ultimately considered clinically non-contributory, underscoring the importance of distinguishing true pathogens from colonization, contamination, or latent viral reactivation.

Diagnostic performance varied substantially according to specimen type. Drainage fluid and abscess specimens yielded the highest clinically meaningful diagnostic contribution rate, whereas plasma and tissue specimens showed relatively lower diagnostic performance. These findings are consistent with previous studies demonstrating that specimens obtained directly from suspected sites of infection generally provide greater clinical utility than plasma in patients with localized infections [5,23]. Therefore, when a potential infectious focus is identified, lesion-directed sampling should be prioritized whenever clinically feasible [24]. Plasma mNGS may serve as a complementary approach, particularly in patients with suspected disseminated infection or without an accessible lesion, or when invasive procedures are contraindicated.

A lower WBC count was independently associated with clinically meaningful mNGS results in our cohort. As the underlying mechanism remains uncertain, this finding should be interpreted cautiously and requires validation in larger prospective studies. One possible explanation is that mNGS may be particularly useful in patients with occult, atypical, or low-grade infections accompanied by relatively limited inflammatory responses.

Consistent with previous studies [5,23], mNGS demonstrated a higher sensitivity but lower specificity than conventional culture for the diagnosis of infectious FUO. However, this finding should be interpreted with caution. Conventional culture has well-recognized limitations in patients with FUO, particularly after prior antimicrobial exposure and for fastidious, intracellular, viral, fungal, or mycobacterial pathogens, which are inherently difficult or impossible to detect in routine cultures. Therefore, the observed difference in sensitivity should not be interpreted as evidence of the absolute superiority of mNGS. Rather, mNGS should be regarded as a complementary diagnostic tool that extends the diagnostic capability of conventional microbiological methods and should be interpreted together with clinical findings and conventional microbiological investigations.

This study has several strengths: we evaluated clinically meaningful diagnostic contributions, rather than positivity alone, and further assessed specimen-specific performance and the impact of mNGS on therapeutic decision-making. However, several limitations should be acknowledged. First, as a single-center retrospective study conducted at a tertiary referral hospital, selection bias is unavoidable, and the findings may not be fully generalizable to all patients with fever of unknown origin. Second, because mNGS results were available to the treating physicians and were considered during the final diagnostic adjudication, incorporation bias cannot be completely excluded, which may have led to an overestimation of the diagnostic contribution of mNGS. Third, although clinically meaningful diagnostic contributions were independently assessed by two experienced infectious disease physicians according to predefined criteria, some degree of subjective judgment remains inevitable. Although disagreements were resolved through consensus discussion, inter-reviewer agreement was not formally assessed because consensus review was prespecified as the adjudication method. Fourth, multiple specimen types were included, and the diagnostic performance of mNGS may vary substantially across different specimen types. Fifth, although mNGS findings led to treatment modification in 57.5% patients with clinically meaningful results, this study was not designed to determine whether these changes translated into improved clinical outcomes. Therefore, we did not evaluate important clinical outcomes such as mortality or disease recurrence, nor did we assess the cost-effectiveness of mNGS. These issues should be addressed in future prospective studies. Sixth, although the event-per-variable ratio was approximately 10 in the multivariable logistic regression analysis, indicating relatively limited model complexity, the possibility of model overfitting could not be completely excluded due to the relatively small sample size and exploratory nature of the analysis. Therefore, the identified association between white blood cell count and clinically meaningful mNGS results should be interpreted cautiously and validated in larger prospective multicenter studies.

Future prospective multicenter studies with standardized diagnostic algorithms, predefined adjudication criteria, and economic evaluations are warranted to further validate the clinical utility of mNGS in patients with fever of unknown origin.

## 5. Conclusions

mNGS provides a clinically meaningful diagnostic contribution in adult patients with fever of unknown origin, particularly by improving the detection of occult and difficult-to-identify infectious etiologies. Its clinical utility extends beyond pathogen identification by facilitating targeted antimicrobial therapy and supporting diagnostic decision-making. Optimized specimen selection, especially lesion-directed sampling when feasible, together with the careful interpretation of sequencing results within the clinical context, is essential to maximize the diagnostic value of mNGS in patients with FUO.

## Figures and Tables

**Table 1:** Baseline characteristics of patients.

Variable	Overall (N = 127)
Age, years	54 (40.0–64.5)
Male	84 (66.1)
Peak body temperature, °C	39.3 ± 0.7
Fever duration, months	2 (1–6)
Length of hospital stay, days	24 (16–32)
Immunocompromised status	22 (17.3)
Comorbidities	
Diabetes mellitus	24 (18.9)
Chronic kidney disease/kidney transplantation	6 (4.7)
Chronic liver disease/viral hepatitis	10 (7.9)
Hematological disorders	4 (3.1)
History of solid malignancy	10 (7.9)
Noninfectious inflammatory diseases	10 (7.9)
White blood cell count (×10^9^/L)	6.66 (4.70–10.27)
Hemoglobin (g/L)	103 (89–116)
Platelet count (×10^9^/L)	281 (174–388)
Erythrocyte sedimentation rate (mm/h)	67 (25–95)
C-reactive protein (mg/L)	60.3 (20.8–124.4)
Procalcitonin (ng/mL)	0.21 (0.09–0.64)
Ferritin (ng/mL)	662 (222–1666)
Interleukin-10 (pg/mL)	5.4 (5.0–14.7)
Positive mNGS findings	72 (56.7)

Data are presented as n (%), mean ± standard deviation (SD), or median (interquartile range [IQR]), as appropriate. Abbreviations: mNGS, metagenomic next-generation sequencing.

**Table 2:** Diagnostic performance of mNGS and conventional culture for identifying infectious etiologies in patients with FUO.

Method	Infectious Disease	Noninfectious Disease	Sensitivity % (95% CI)	Specificity % (95% CI)	PPV % (95% CI)	NPV % (95% CI)
mNGS Positive	47	25	69.1 (56.7, 79.8)	57.6 (44.1, 70.4)	65.3 (53.1, 76.1)	61.8 (47.7, 74.6)
mNGS Negative	21	34	—	—	—	—
Culture Positive	12	1	16.9 (9.0, 27.7)	96.0 (79.6, 99.9)	92.3 (64.0, 99.8)	28.9 (19.5, 39.9)
Culture Negative	59	24	—	—	—	—

Conventional culture results were available for 96 patients; therefore, diagnostic performance metrics for culture were calculated based on patients with available culture results. Abbreviations: mNGS, metagenomic next-generation sequencing; PPV, positive predictive value; NPV, negative predictive value; FUO, fever of unknown origin.

**Table 3:** Comparison of baseline characteristics between patients with and without clinically meaningful diagnostic contribution of mNGS.

Variable	Clinically Meaningful: Diagnostic Contribution Group (n = 40)	Not Clinically Meaningful: Diagnostic Contribution Group (n = 87)	*p* Value
Age, years	46.8 ± 16.1	53.8 ± 16.3	0.03
Male	25 (62.5)	59 (67.8)	0.56
Peak body temperature, °C	39.4 ± 0.8	39.2 ± 0.8	0.21
Duration of fever, months	1.5 (1.0–6.5)	2.0 (1.0–6.0)	0.83
Length of hospital stay, days	27.2 ± 11.4	25.6 ± 13.5	0.52
Immunocompromised status	6 (15.0)	16 (18.4)	0.56
Diabetes mellitus	7 (17.5)	17 (19.5)	0.79
White blood cell count (×10^9^/L)	6.8 ± 3.5	9.4 ± 6.8	0.01
Hemoglobin (g/L)	101 ± 23	104 ± 30	0.66
Platelet count (×10^9^/L)	267 ± 107	296 ± 166	0.28
Erythrocyte sedimentation rate (mm/h)	51 ± 36	61 ± 34	0.19
C-reactive protein (mg/L)	92 ± 98	76 ± 61	0.41
Procalcitonin (ng/mL)	0.26 (0.10–0.75)	0.25 (0.14–0.68)	0.99
Ferritin (ng/mL)	347 (171–829)	519 (225–1212)	0.36
Interleukin-10 (pg/mL)	5.0 (5.0–33.5)	5.7 (5.0–13.9)	0.85
ALT (U/L)	32 ± 26	42 ± 49	0.15
AST (U/L)	31 ± 17	46 ± 58	0.047
Total bilirubin (μmol/L)	11 ± 6	12 ± 8	0.57
Albumin (g/L)	36 ± 6	35 ± 5	0.33
Serum creatinine (μmol/L)	85 ± 101	70 ± 34	0.42

Data are presented as n (%), mean ± standard deviation (SD), or median (interquartile range [IQR]), as appropriate. Abbreviations: mNGS, metagenomic next-generation sequencing;; ALT, alanine aminotransferase; AST, aspartate aminotransferase.

**Table 4:** Multivariable logistic regression analysis of factors associated with clinically meaningful diagnostic contribution of mNGS.

Variable	Coefficient (β)	OR (95% CI)	*p* Value
Age	−0.03	0.97 (0.94–1.00)	0.06
WBC	−0.14	0.87 (0.77–0.98)	0.02
AST	−0.03	0.98 (0.95–1.00)	0.06
PCT	−0.01	0.99 (0.97–1.02)	0.55

Abbreviations: mNGS, metagenomic next-generation sequencing; WBC, white blood cell count; AST, aspartate aminotransferase; PCT, procalcitonin; OR, odds ratio; CI, confidence interval.

## Data Availability

The data presented in this study are available from the corresponding author upon reasonable request. The data are not publicly available because of patient privacy and institutional regulations.

## References

[1] Wright W.F., Durso S.C., Forry C., Rovers C.P. (2025). Fever of unknown origin. BMJ.

[2] Kang S., Zheng R. (2024). Distribution of the causes of fever of unknown origin in China, 2013–2022. J. Transl. Int. Med..

[3] Mourad O., Palda V., Detsky A.S. (2003). A comprehensive evidence-based approach to fever of unknown origin. Arch. Intern. Med..

[4] Fusco F.M., Pisapia R., Nardiello S., Cicala S.D., Gaeta G.B., Brancaccio G. (2019). Fever of unknown origin (FUO): Which are the factors influencing the final diagnosis? A 2005-2015 systematic review. BMC Infect. Dis..

[5] Chiu C.Y., Miller S.A. (2019). Clinical metagenomics. Nat. Rev. Genet..

[6] Miao Q., Ma Y., Wang Q., Pan J., Zhang Y., Jin W., Yao Y., Su Y., Huang Y., Wang M. (2018). Microbiological Diagnostic Performance of Metagenomic Next-generation Sequencing When Applied to Clinical Practice. Clin. Infect. Dis..

[7] Zhao Y., Zhang W., Zhang X. (2024). Application of metagenomic next-generation sequencing in the diagnosis of infectious diseases. Front. Cell Infect. Microbiol..

[8] Zhang P., Liu B., Zhang S., Chang X., Zhang L., Gu D., Zheng X., Chen J., Xiao S., Wu Z. (2024). Clinical application of targeted next-generation sequencing in severe pneumonia: A retrospective review. Crit. Care.

[9] Wang W., Chauhan V., Luo Y., Sharma S., Li C., Chen H. (2024). Comparing NGS-Based identification of bloodstream infections to traditional culture methods for enhanced ICU care: A comprehensive study. Front. Cell Infect. Microbiol..

[10] Liu J., Dong Y., Huang Y., Xie M., Wang H., Wang Q., Wang S., Wang N., Jiang Y., Zhang W. (2025). Clinical metagenomic next-generation sequencing test for diagnosis of central nervous system infections in ICU: A multicenter retrospective study. Int. J. Infect. Dis..

[11] Fu Z.F., Zhang H.C., Zhang Y., Cui P., Zhou Y., Wang H.Y., Lin K., Zhou X., Wu J., Wu H.L. (2021). Evaluations of Clinical Utilization of Metagenomic Next-Generation Sequencing in Adults With Fever of Unknown Origin. Front. Cell Infect. Microbiol..

[12] Dong Y., Gao Y., Chai Y., Shou S. (2022). Use of Quantitative Metagenomics Next-Generation Sequencing to Confirm Fever of Unknown Origin and Infectious Disease. Front. Microbiol..

[13] Daher M., Iordanov R., Al Mohajer M., Sohail M.R., Staggers K.A., Hamdi A.M. (2024). Clinical utility of metagenomic next-generation sequencing in fever of undetermined origin. Ther. Adv. Infect. Dis..

[14] Mao J.Y., Li D.K., Zhang D., Yang Q.W., Long Y., Cui N. (2024). Utility of paired plasma and drainage fluid mNGS in diagnosing acute intra-abdominal infections with sepsis. BMC Infect. Dis..

[15] Marra A.R., Lopes G.O.V., Pardo I., Hsieh M.K., Kobayashi T., Marra P.S., Marschall J., Pinho J.R.R., Amgarten D.E., de Mello Malta F. (2024). Metagenomic next-generation sequencing in patients with fever of unknown origin: A comprehensive systematic literature review and meta-analysis. Diagn. Microbiol. Infect. Dis..

[16] Blauwkamp T.A., Thair S., Rosen M.J., Blair L., Lindner M.S., Vilfan I.D., Kawli T., Christians F.C., Venkatasubrahmanyam S., Wall G.D. (2019). Analytical and clinical validation of a microbial cell-free DNA sequencing test for infectious disease. Nat. Microbiol..

[17] Xie N., Zhang W., Tian F., Sun W., Xing M., Ruan Q., Song J. (2025). Fever of unknown origin: Clinical significance of the etiology and common inflammatory parameters. Diagn. Microbiol. Infect. Dis..

[18] Wright W.F., Mulders-Manders C.M., Auwaerter P.G., Bleeker-Rovers C.P. (2022). Fever of Unknown Origin (FUO)—A Call for New Research Standards and Updated Clinical Management. Am. J. Med..

[19] Boumaza A., Ben Azzouz E., Arrindell J., Lepidi H., Mezouar S., Desnues B. (2022). Whipple’s disease and *Tropheryma whipplei* infections: From bench to bedside. Lancet Infect. Dis..

[20] Abedi Z., Sheikh Beig Goharrizi M.A., Abbasi A., Sadat Soleimani Zakeri N., Jangi H. (2025). Metagenomic insights into microbial community alterations and co-occurrence networks in infective endocarditis. Genom. Inf..

[21] Kumari I., Lakhanpal D., Swargam S., Nath Jha A. (2023). Leishmaniasis: Omics Approaches to Understand its Biology from Molecule to Cell Level. Curr. Protein Pept. Sci..

[22] Xing F., Deng C., Luo Z., Tsang C.C., Lau S.K.P., Woo P.C.Y. (2025). Emergence of Next-Generation Sequencing for Laboratory Diagnosis of *Talaromyces marneffei*. Mycopathologia.

[23] Gu W., Miller S., Chiu C.Y. (2019). Clinical Metagenomic Next-Generation Sequencing for Pathogen Detection. Annu. Rev. Pathol..

[24] Wilson M.R., Sample H.A., Zorn K.C., Arevalo S., Yu G., Neuhaus J., Federman S., Stryke D., Briggs B., Langelier C. (2019). Clinical Metagenomic Sequencing for Diagnosis of Meningitis and Encephalitis. N. Engl. J. Med..

